# The anteroposterior femoral translation starting angle and the medial pivot pattern are correlated with the range of motion after total knee arthroplasty

**DOI:** 10.1002/jeo2.70435

**Published:** 2025-09-09

**Authors:** Ryota Takase, Shogo Hashimoto, Takashi Ohsawa, Hibiki Kakiage, Akira Honda, Hirotaka Chikuda

**Affiliations:** ^1^ Department of Orthopaedic Surgery Gunma University Graduate School of Medicine Maebashi Gunma Japan

**Keywords:** anteroposterior femoral translation starting angle, image‐less navigation, medial pivot pattern, posterior‐stabilized total knee arthroplasty, range of motion

## Abstract

**Purpose:**

Postoperative range of motion (ROM) of the knee is an important factor for improving clinical scores and symptoms in total knee arthroplasty (TKA). This study aimed to investigate the relationship between intraoperative factors, including anteroposterior translation of the femur, and extension and flexion gaps, observed during posterior‐stabilized TKA (PSTKA) using a navigation system, and preoperative and postoperative parameters.

**Methods:**

Twenty‐one knees with osteoarthritis that were treated by PSTKA were included. ROM and clinical outcomes, including the Hospital for Special Surgery Knee Score (HSS score), were measured before and one year after surgery, and intraoperative kinematic factors were measured using an image‐less navigation system. We further divided the subjects into two groups based on the presence or absence of postoperative flexion contracture (defined as an extension angle ≤ –5°at one year after surgery). The no contracture group comprised 12 knees, and the contracture group comprised 9 knees.

**Results:**

The postoperative extension angle correlated with the starting angle of the anteroposterior translation of the femur (*r* = –0.60; *p* < 0.01) and the postoperative HSS score (*r* = 0.46; *p* = 0.04). The postoperative flexion angle correlated with the lateral‐to‐medial anteroposterior translation ratio (*r* = 0.47; *p* = 0.03), indicating a medial pivot pattern. In comparison to the contracture group, the no contracture group had a significantly smaller starting angle of the anteroposterior translation of the femur in comparison to the contracture group (no contracture, 30.6° ± 17.0°; contracture, 48.4° ± 16.7°; *p* = 0.02) and a significantly higher postoperative HSS score (no contracture, 90.8 ± 7.0; contracture, 81.1 ± 11.8; *p* < 0.05).

**Conclusions:**

This study revealed that knee joints with flexion contracture after PSTKA had a significantly larger starting angle of anteroposterior translation during surgery and that an intraoperative medial pivot pattern was beneficial for postoperative flexion angles.

**Level of Evidence:**

Level III.

**Abbreviations**
FJS: Forgotten Joint Score
HKA: hip‐knee‐ankle
HSS: hospital for special surgery
ICC: intraclass correlation coefficient
PS: posterior‐stabilized
ROM: range of motion
TKA: total knee arthroplasty

## INTRODUCTION

Total knee arthroplasty (TKA) is effective for relieving pain and improving the knee function of osteoarthritic knees [30]. The range of motion (ROM) after TKA affects patient satisfaction and functional activities after surgery [21, 22]. The persistence of flexion contracture leads to pain [7]. One of the important outcomes of TKA is the restoration of full extension; patients with flexion contracture have poor postoperative outcomes, and the degree of flexion contracture is correlated with poor postoperative outcomes [26, 28]. While there is no restriction of ROM during or immediately after TKA, joint contracture resulting from the effects of soft tissue, including arthrofibrosis, may occur in the postoperative period [3, 14]. Previous studies have shown that the preoperative ROM and the amount of gap in extension and flexion during surgery affect postoperative ROM [24, 26].

During normal knee flexion, anteroposterior femoral translation contributes to flexion, and various factors including alignment can cause a medial pivot pattern, one of the characteristics of this is that the lateral femoral condyle moves posteriorly relative to the medial femoral condyle [2, 12, 23]. Therefore, anteroposterior translation of the femur is an important factor in knee kinematics, including a favorable ROM and an image‐less navigation system enables the examination of detailed data on the angle at which the femur begins to move posteriorly and the amount of femur translation, and extension and flexion gaps [2, 5, 35]. Image‐less navigation allows knee kinematics to be evaluated during surgery without interference from soft tissue through identification of the skeletal structure [4, 27]. In addition, the ratio of the amount of translation of the medial femoral condyle to that of the lateral femoral condyle can be calculated to investigate the medial pivot pattern [15].

This study evaluated the correlation between ROM after TKA, preoperative ROM, the extension gap, the 90° flexion gap, the starting angle of the anteroposterior translation of the femur, and the lateral‐to‐medial anterior‐posterior translation ratio using an image‐less navigation system in detail. We hypothesized that the postoperative ROM is affected by the intraoperative starting angle of the anteroposterior translation of the femur.

## MATERIALS AND METHODS

### Subjects

**Figure 1:**
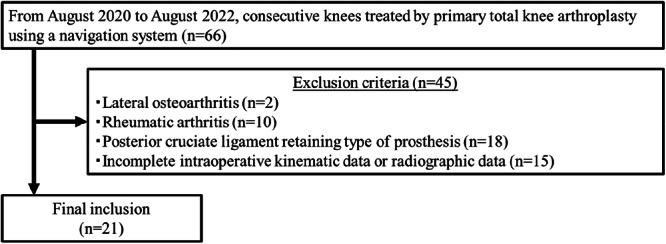
Detailing the study enrollment process.

Approval from the Ethical Review Board was obtained for this study (approval number was HS2021‐170). This retrospective study included consecutive 66 knees (60 patients) treated by primary TKA using an image‐less navigation system at our hospital from August 2020 to August 2022. Informed consent was obtained from all patients included in the study. The following exclusion criteria were applied: lateral osteoarthritis (*n* = 2), rheumatic arthritis (*n* = 10), posterior cruciate ligament retaining type of prosthesis (*n* = 18), and incomplete intraoperative kinematic or radiographic data (*n* = 15). After applying the exclusion criteria, the remaining 21 knees (20 patients) with Kellgren–Lawrence Grade 4 osteoarthritis were included in the study (Figure 1). Patients who had previously undergone arthroscopic surgery were included, while those who had undergone other procedures, such as osteotomy, were not included. There were no patients with severe varus deformities (> 20°). For all knees, the knee ROM was measured with a goniometer both before surgery and 1 year after surgery [36]. The patients were divided into the no contracture group (without flexion contracture [extension angle > –5°]) and the contracture group (flexion contracture [extension angle ≤ –5°]) based on the extension angle 1 year postoperatively [25]. The Hospital for Special Surgery Knee Score (HSS score) was measured before surgery and 1 year after surgery, and the Forgotten Joint Score (FJS) was measured 1 year after surgery to assess clinical outcomes. Preoperative and postoperative radiographs were also obtained. The hip‐knee‐ankle angle (HKA) and the posterior tibial slope angle were measured (Table 1).

**Table 1:** Demographic data.

Age (years)	74.3 ± 6.5
Sex (male/female)	3/18
BMI (kg/m²)	26.8 ± 3.1
Side (right/left)	9/12
Preoperative HKA (°)	12.1 ± 5.3
Preoperative posterior tibial slope angle (°)	9.2 ± 4.1
Preoperative extension angle (°)	–11.4 ± 7.7
Preoperative flexion angle (°)	117.4 ± 18.0
Preoperative HSS score	58.3 ± 12.1

*Note*: Values represent the mean ± standard deviation.

Abbreviations: BMI, body mass index; HKA, hip‐knee‐ankle angle; HSS score, Hospital for Special Surgery Knee Score.

### Surgical procedures

Posterior‐stabilized (PS) type TKA (Attune, DePuy Synthes, Warsaw, Indiana, USA) was used and the operation was performed using an image‐less navigation system (Knee3, BrainLab, Germany, Munich). Surgery was performed using a medial parapatellar approach and a measured resection technique with an air tourniquet under general anesthesia. None of the patients had undergone patellar surface replacement surgery. Several orthopedic surgeons at the hospital performed all surgeries. After an incision was made in the knee joint capsule, two cortical screws were inserted into the femur and tibia and passive optical reference arrays were attached to identify the orientation. The registration was carried out in the same way as in a previous study [34]. Briefly, after identifying the hip center, the main landmarks (the medial and lateral malleoli; proximal tibia and distal femur mechanical axis; medial, lateral, and anterior proximal tibial contour; medial and lateral epicondyles; and femoral anterior sizing point) were registered by touching the appropriate bony structures. Proximal tibial osteotomy was performed perpendicular to the mechanical axis in the coronal plane and with a 3° tibial posterior inclination in the sagittal plane. Tibial rotation was determined using the Akagi line (from the posterior cruciate ligament insertion to the medial edge of the tibial tuberosity) [1].

### Image‐less navigation system

**Figure 2:**
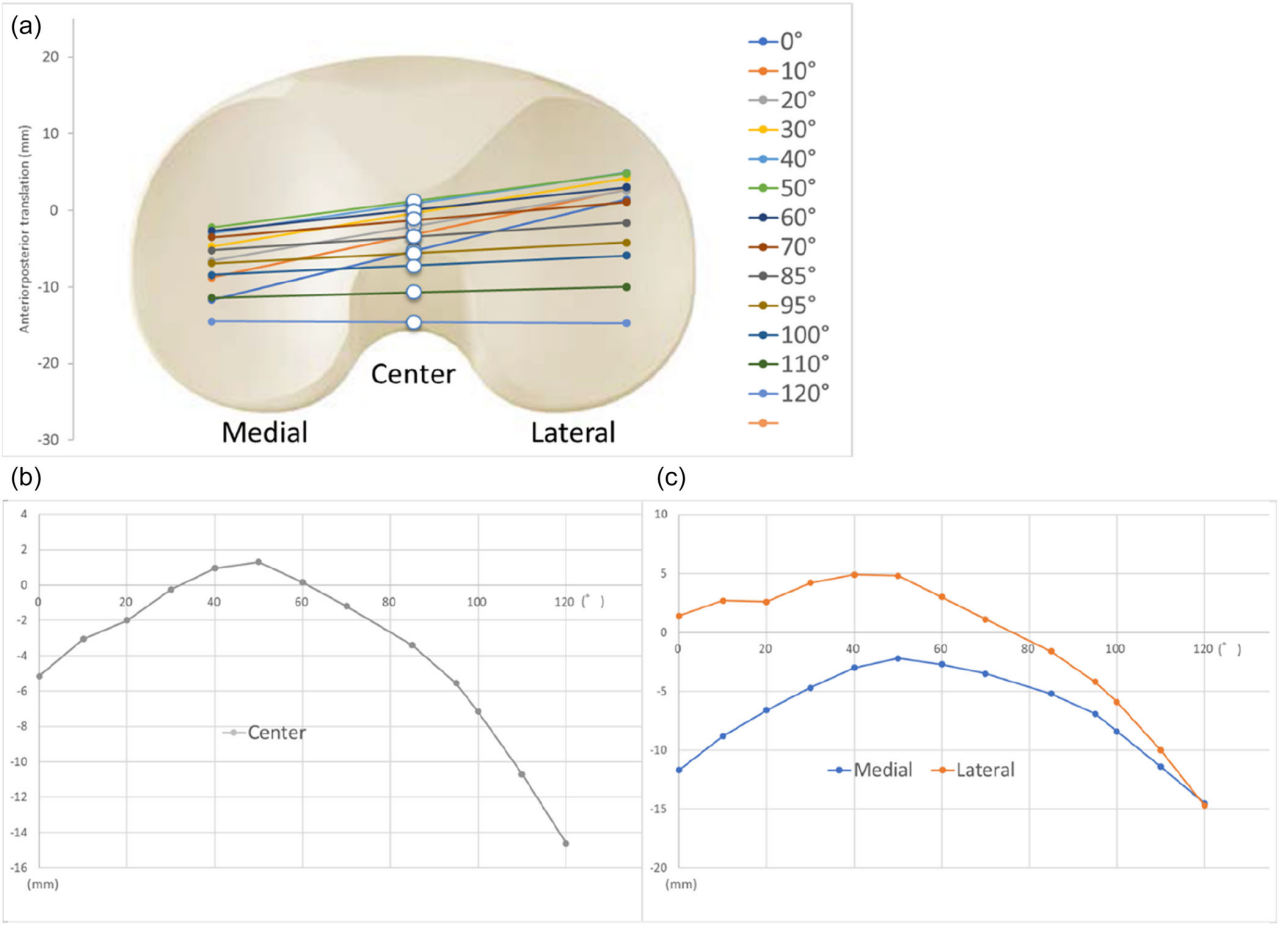
Anteroposterior translation of the femur relative to the tibia at each flexion angle. (a) The lines indicate the femoral condylar axis. (b) Anteroposterior translation of the femoral center. (c) Anteroposterior translation of the medial and lateral femoral condyles.

Knee kinematics, including stability and alignment during surgery after implant insertion and ligament tensioner placement were investigated. Passive ROM from a completely extended leg to full extension and full flexion without stress was recorded [34]. Figure 2 presents the navigation data showing the anteroposterior movement of the femur relative to the tibia at each degree of knee flexion during the surgery. The anteroposterior translation of the medial and lateral femoral condyles relative to the tibial axis was measured. Anteroposterior translation of the femur was expressed as a percentage, calculated by dividing the amount of anteroposterior translation of the femur by the anteroposterior length of the tibial tray [15]. The anteroposterior translation of the femur was measured with the patella restored, and the implant was placed in position. The medial and lateral joint gaps at the extension and 90° flexion positions were measured with a force of 113 N in each compartment [35]. When the origin of the femur coordinate system was posterior to the origin of the tibia coordinate system, the anteroposterior translation was determined to be “+” and when the origin of the femur coordinate system was anterior to the origin of the tibia coordinate system, it was determined to be “–”. The starting angle was defined as the knee flexion angle at which the center of the femoral condyles began to move posteriorly during anteroposterior translation of the femur (Figure 2). Additionally, the lateral‐to‐medial anteroposterior translation ratio was defined as lateral anteroposterior translation divided by medial anteroposterior translation [15].

### Statistical analysis

The Shapiro–Wilk test was used to assess the normality of all data. As non‐normal distributions were observed both preoperatively and postoperatively, Spearman's correlation coefficients were calculated. The Mann–Whitney *U* test was used to determine the statistical significance of the differences between the two groups (with or without flexion contracture). The effect size was reported using Hedges' g. Continuous variables are expressed as the mean ± standard deviation.

The inter‐ and intra‐rater reliabilities of ROM measurements were assessed using the intraclass correlation coefficient (ICC). Two orthopedic surgeons independently measured ROM in a blinded manner. To calculate inter‐ and intra‐rater reliability, a single observer examined 10 measurements twice at 6‐week intervals. Knee extension measurements had an intraclass reliability of 0.851 and interclass reliability of 0.816. The knee flexion measurements had an intraclass reliability of 0.982 and an interclass reliability of 0.745. All statistical analyses were performed using SPSS (ver. 29.0 IBM Corp., Armonk, NY, USA). Statistical significance was set at *p* < 0.05.

## RESULTS

Twenty‐one knees with osteoarthritis that were treated by PSTKA using an image‐less navigation system were included in the study. The mean age of the patients was 74.3 ± 6.5 years. Eighteen (86%) participants were female. The postoperative extension angle after 1 year was moderately correlated with the preoperative extension angle (*r* = 0.47; *p* = 0.03), preoperative flexion angle (*r* = 0.44; *p* < 0.05), starting angle of the anteroposterior translation of the femur (*r* = –0.60; *p* < 0.01), and postoperative HSS score (*r* = 0.46; *p* = 0.04). The postoperative flexion angle after 1 year was moderately correlated with the preoperative flexion angle (*r* = 0.51; *p* = 0.02) and lateral‐to‐medial anteroposterior translation ratio (*r* = 0.47; *p* = 0.03). The FJS was moderately correlated with the postoperative HSS score (*r* = 0.50; *p* = 0.02). The postoperative HSS score was moderately correlated with age (*r* = –0.58; *p* < 0.01) and preoperative HKA (*r* = –0.44; *p* < 0.05).

**Figure 3:**
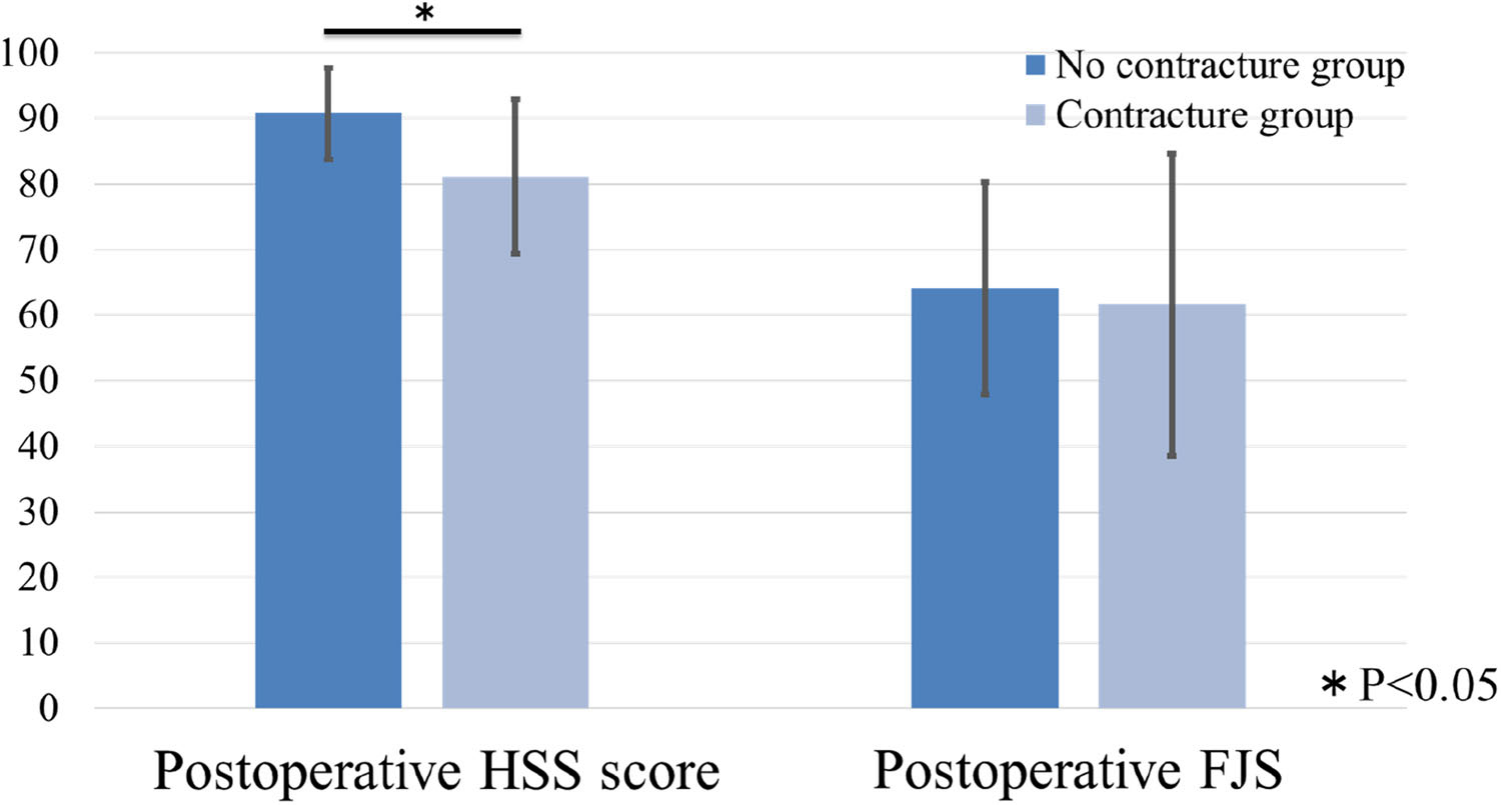
Comparison of postoperative clinical outcomes between no contracture and contracture groups. FJS, Forgotten Joint Score; HSS score, Hospital for Special Surgery Knee Score. *p < 0.05.

According to the postoperative extension angle after 1 year, 12 knees were classified into the no contracture group, and 9 knees were classified into the contracture group. Significant differences were found between the two groups in the preoperative extension angle (–8.3° ± 6.6° for the no contracture group, –15.6° ± 7.2° for the contracture group; *p* = 0.04) (Table 2). The no contracture group had a significantly smaller starting angle of the anteroposterior translation of the femur in comparison to the contracture group (30.6° ± 17.0° vs. 48.4° ± 16.7°; *p* = 0.02) (Table 3). The no contracture group had a significantly higher postoperative HSS score after 1 year in comparison to the contracture group (90.8 ± 7.0 vs. 81.1 ± 11.8; *p* < 0.05) (Figure 3). The two groups showed no significant differences in the gap between medial and lateral extension and flexion (Table 4).

**Table 2:** Demographic data in no contracture and contracture groups.

	No contracture group (*n* = 12)	Contracture group (*n* = 9)	Effect size	*p*‐value
Age (years)	72.7 ± 4.6	76.6 ± 7.8	0.20	0.27
BMI (kg/m²)	26.5 ± 2.7	27.3 ± 3.5	0.11	0.52
Preoperative HKA (°)	11.0 ± 5.1	13.7 ± 5.2	0.29	0.13
Preoperative posterior tibial slope angle (°)	9.3 ± 3.9	9.2 ± 4.4	0.04	0.83
Preoperative extension angle (°)	–8.3 ± 6.6	–15.6 ± 7.2	0.40	0.04*
Preoperative flexion angle (°)	122.9 ± 16.9	110.0 ± 16.8	0.38	0.05
Preoperative HSS score	60.7 ± 10.8	55.2 ± 12.9	0.17	0.36

*Note*: Values represent the mean ± standard deviation.

Abbreviations: BMI, body mass index; HKA, hip‐knee‐ankle angle; HSS score, Hospital for Special Surgery Knee Score.

* *p* < 0.05.

**Table 3:** Comparison of parameters during surgery between no contracture and contracture groups.

	No contracture group (*n* = 12)	Contracture group (*n* = 9)	Effect size	*p*‐value
Anteroposterior translation of the femur (%)	26.9 ± 16.7	20.9 ± 13.1	0.13	0.48
Starting angle of the anteroposterior translation (°)	30.6 ± 17.0	48.4 ± 16.7	0.46	0.02*
Lateral‐to‐medial anterior‐posterior translation ratio	5.3 ± 11.9	1.3 ± 3.9	0.01	0.94
Medial joint gap at extension (mm)	10.5 ± 2.2	11.4 ± 2.7	0.18	0.35
Lateral joint gap at extension (mm)	12.6 ± 2.6	13.1 ± 2.9	0.09	0.65
Medial joint gap at 90° flexion (mm)	11.6 ± 2.8	12.9 ± 1.5	0.20	0.28
Lateral joint gap at 90° flexion (mm)	13.5 ± 3.0	14.3 ± 2.4	0.20	0.75

*Note*: Values represent the mean ± standard deviation.

* *p* < 0.05.

**Table 4:** Comparison of postoperative clinical outcomes between no contracture and contracture groups.

	No contracture group (*n* = 12)	Contracture group (*n* = 9)	Effect size	*p*‐value
Postoperative HKA (°)	–0.1 ± 1.7	1.7 ± 3.1	0.26	0.17
Postoperative posterior tibial slope angle (°)	1.4 ± 1.3	1.8 ± 1.8	0.07	0.71
Postoperative extension angle (°)	0.0 ± 0.0	−8.3 ± 3.3	2.13	<0.01*
Postoperative flexion angle (°)	124.2 ± 10.2	118.9 ± 14.3	0.12	0.51
Postoperative HSS score	90.8 ± 7.0	81.1 ± 11.8	0.39	0.05*
Postoperative FJS	64.1 ± 16.2	61.6 ± 23.1	0.01	0.94

*Note*: Values represent the mean ± standard deviation.

Abbreviations: HKA, hip‐knee‐ankle angle; FJS, Forgotten Joint Score; HSS score, Hospital for Special Surgery Knee Score.

* *p* < 0.05.

## DISCUSSION

This study has two important findings. First, the extension angle after PSTKA is associated with the starting angle of the anteroposterior translation of the femur during surgery. Second, the flexion angle after PSTKA was larger in knees with a medial pivot pattern. However, the appropriately adjusted extension and flexion gaps during surgery did not affect the postoperative ROM.

### The anteroposterior translation of the femur

In knee joints with PSTKA, femoral rollback occurs due to the engagement of the cam‐post, which engages at a flexion angle of 60°–90° [19, 32]. However, the initiation angle of the femoral rollback is below the expected flexion angle of the cam‐post engagement [4]. In the present study, the femur began to move posteriorly at an average angle of 38.3° (< 60°). This result is in line with previous studies [4, 32, 35]. In a knee joint without cruciate ligaments, the femur begins to move posteriorly based on the geometry of the articulating surfaces and the remaining soft tissue structures, and a similar mechanism is thought to occur during passive motion in PSTKA [4]. This mechanism might indicate that the posterior soft tissue begins to become lax owing to knee flexion before cam‐post engagement, causing the femur to begin moving posteriorly. In addition, even if the knee is fully extended during surgery, postoperative flexion contracture is thought to be caused after TKA because of posterior soft tissue contracture and loss of extensibility [16]. Therefore, the results of the present study indicate that stiffness of the posterior soft tissue can be evaluated during PSTKA surgery by measuring the starting angle of the anteroposterior translation of the femur. In the present study, the starting angle of the anteroposterior translation of the femur was significantly smaller (30.6° ± 17.0°) in the knee joint without flexion contracture after surgery. This suggests that the posterior soft tissues may have been laxer than the knee joint with extension restriction after surgery.

### The medial pivot pattern

A larger lateral rollback and medial pivoting pattern is known as a normal knee kinematic pattern [5, 33]. However, this kinematic pattern may not be observed in patients with knee osteoarthritis, and not only the skeletal structure but also soft tissues such as ligaments and muscles are related to knee kinematics [2, 23]. In comparison to TKA implant designs that retain the posterior cruciate ligament, the distance of rollback of the femoral medial and lateral condyles tends to be similar in PSTKA [5, 15]. However, because the surrounding soft tissue will also have an effect. In the present study, knee joints with PSTKA that induced medial pivot patterns after surgery were also observed. In addition, previous studies that analyzed PSTKA kinematics revealed that the postoperative medial pivot kinematic pattern was observed in 46%–57% of cases [5, 29, 34]. In the present study, a correlation was observed between the medial pivot pattern during PSTKA surgery and the postoperative flexion angle, revealing that the flexion angle was larger. This result indicates that the flexion angle increased after PSTKA in the knee joint with a restored physiological kinematic pattern. When anatomy is reconstructed after surgery, normal kinematics are preserved [9], and the knee joint may induce medial pivot patterns after surgery. The optimal technique for restoring a medial pivot pattern in PSTKA remains unclear; however, current implant designs that promote medial pivot patterns may prove effective. On the other hand, the survivability of new implant designs, including medial pivot designs, remains uncertain [18].

### The effect of ROM after surgery on clinical outcomes

Postoperative ROM after TKA affects patient satisfaction [21, 22]. In the present study, the HSS score after TKA was correlated with the postoperative extension angle, and the postoperative HSS score was correlated with postoperative FJS. Patients with knee joints that have flexion contracture after TKA have lower postoperative satisfaction and walking ability [8, 26]. There are problems associated with mild knee flexion contracture, which is known to increase muscular demand and associated physiological responses during locomotion [13, 20]. In addition, flexion contracture in the operated knee can also lead to mechanical strain on the contralateral knee and worsening of disease progression [11]. However, some studies have reported that there is no correlation between flexion angle and patient‐reported outcome measures [6, 31]. Conversely, some studies have reported that the flexion angle after surgery is correlated with patient‑reported outcome measures and affects satisfaction [10, 17]. The postoperative flexion angle is a controversial parameter that affects postoperative clinical scores; however, since better knee ROM may lead to a better knee function, postoperative ROM is considered to be an important factor that should be targeted to improve clinical outcomes and symptoms after TKA.

The present study was associated with several limitations. First, the posterior condylar offset of the femur was not evaluated. However, methods for evaluating the offset of the posterior condyle of the femur have not yet been standardized. Second, as we evaluated the ROM one year after surgery, the long‐term effects remain uncertain. However, while one year may be relatively short to evaluate postoperative outcomes, we considered this period reasonable because previous studies showed that postoperative ROM reaches a plateau one year after surgery [36]. Third, this study only evaluated the PSTKA. Fourth, the number of cases included in this study was small, which has limited statistical power, especially for parameters without significant differences. Future studies with larger sample sizes are warranted to validate our findings. Fifth, intraoperative evaluation involved passive movement under anesthesia. Sixth, multiple surgeons performed the procedure using the same concept and techniques; despite this standardization, inter‑surgeon variability may have affected the outcome. Seventh, the lateral‐to‐medial anteroposterior translation ratio represents only one characteristic of the medial pivot pattern and is not an absolute indicator of medial pivot. Eighth, postoperative pain and rehabilitation that may influence postoperative ROM were not included in this study and should be incorporated into future analyses. Ninth, a history of arthroscopic surgery was considered a potential confounding factor that might have influenced intraoperative kinematics and postoperative outcomes.

The clinical relevance of the present study is that analyzing the starting angle of the anteroposterior translation and medial pivot patterns during surgery in PSTKA can potentially predict postoperative knee joint ROM. In addition, the translation of the femur during TKA more closely matches that of a normal knee, which allows for a better ROM after surgery.

## CONCLUSION

Although the sample size of this study was limited, the findings suggest that the starting angle of anteroposterior translation of the femur and the medial pivot pattern observed during PSTKA are correlated with postoperative range of motion. Further studies with larger sample sizes are needed to clarify the relationship between intraoperative kinematics and postoperative outcomes.

## AUTHOR CONTRIBUTIONS

Ryota Takase designed the study. Ryota Takase carried out data analysis and wrote the manuscript. Takashi Ohsawa and Shogo Hashimoto helped to design this study. Hibiki Kakiage helped to evaluate items. Hirotaka Chikuda, Takashi Ohsawa, Shogo Hashimoto, and Akira Honda checked the study and helped to modify the manuscript. All authors read and approved of the final manuscript.

## CONFLICT OF INTEREST STATEMENT

The authors declare no conflict of interest.

## ETHICS STATEMENT

The present study was ethically approved by the ethical review board of the hospital where the study was performed (approval number was HS2021‐170). Informed consent was obtained from all patients included in the study.

## Data Availability

The authors have nothing to report.

## References

[1] Akagi M , Oh M , Nonaka T , Tsujimoto H , Asano T , Hamanishi C . An anteroposterior axis of the tibia for total knee arthroplasty. Clin Orthop Relat Res. 2004;420:213–219. 10.1097/00003086-200403000-00030 15057100

[2] Asano T , Akagi M , Tanaka K , Tamura J , Nakamura T . In vivo three‐dimensional knee kinematics using a biplanar image‐matching technique. Clin Orthop Relat Res. 2001;388:157–166. 10.1097/00003086-200107000-00023 11451115

[3] Cartwright‐Terry M , Cohen DR , Polydoros F , Davidson JS , Santini AJ . Manipulation under anaesthetic following total knee arthroplasty: predicting stiffness and outcome. J Orthop Surg. 2018;26:2309499018802971. 10.1177/2309499018802971 30270788

[4] Cromie MJ , Siston RA , Giori NJ , Delp SL . Posterior cruciate ligament removal contributes to abnormal knee motion during posterior stabilized total knee arthroplasty. J Orthop Res. 2008;26:1494–1499. 18464260 10.1002/jor.20664 PMC5507205

[5] Dennis DA , Komistek RD , Mahfouz MR , Haas BD , Stiehl JB . Multicenter determination of in vivo kinematics after total knee arthroplasty. Clin Orthop Relat Res. 2003:37–57. 10.1097/01.blo.0000092986.12414.b5 14646738

[6] Devers BN , Conditt MA , Jamieson ML , Driscoll MD , Noble PC , Parsley BS . Does greater knee flexion increase patient function and satisfaction after total knee arthroplasty? J Arthroplasty. 2011;26:178–186. 20413247 10.1016/j.arth.2010.02.008

[7] Fehring TK , Odum SM , Griffin WL , McCoy TH , Masonis JL . Surgical treatment of flexion contractures after total knee arthroplasty. J Arthroplasty. 2007;22:62–66. 17823018 10.1016/j.arth.2007.03.037

[8] Goudie ST , Deakin AH , Ahmad A , Maheshwari R , Picard F . Flexion contracture following primary total knee arthroplasty: risk factors and outcomes. Orthopedics. 2011;34:e855–e859. 22146201 10.3928/01477447-20111021-18

[9] Graichen H , Avram GM , Strauch M , Kaufmann V , Hirschmann MT . Tibia‐first, gap‐balanced patient‐specific alignment restores bony phenotypes and joint line obliquity in a great majority of varus and straight knees and normalises valgus and severe varus deformities. Knee Surg Sports Traumatol Arthrosc. 2024;32:1287–1297. 38504509 10.1002/ksa.12145

[10] Ha CW , Park YB , Song YS , Kim JH , Park YG . Increased range of motion is important for functional outcome and satisfaction after total knee arthroplasty in asian patients. J Arthroplasty. 2016;31:1199–1203. 26777578 10.1016/j.arth.2015.12.018

[11] Harato K , Nagura T , Matsumoto H , Otani T , Toyama Y , Suda Y . Extension limitation in standing affects weight‐bearing asymmetry after unilateral total knee arthroplasty. J Arthroplasty. 2010;25:225–229. 19264442 10.1016/j.arth.2009.02.003

[12] Johal P , Williams A , Wragg P , Hunt D , Gedroyc W . Tibio‐femoral movement in the living knee. A study of weight bearing and non‐weight bearing knee kinematics using ‘interventional’ MRI. J Biomech. 2005;38:269–276. 15598453 10.1016/j.jbiomech.2004.02.008

[13] Jung MC , Park D , Lee SJ , Lee KS , Kim DM , Kong YK . The effects of knee angles on subjective discomfort ratings, heart rates, and muscle fatigue of lower extremities in static‐sustaining tasks. Appl Ergon. 2010;42:184–192. 20723884 10.1016/j.apergo.2010.07.004

[14] Karam MD , Pugely A , Callaghan JJ , Shurr D . Hinged cast brace for persistent flexion contracture following total knee replacement. Iowa Orthop J. 2011;31:69–72. 22096423 PMC3215117

[15] Kim J , Park JH , Park JH , Son DW , Ahn JH . Prospective sequential comparison of femoral roll‐back between cruciate‐retaining and posterior‐stabilized total knee arthroplasty using an intra‐operative sensor. Knee. 2022;39:253–260. 36283283 10.1016/j.knee.2022.09.013

[16] Kim SH , Ro DH , Cho Y , Lee YM , Lee S , Lee MC . What is the ideal degree of extension after primary total knee arthroplasty? J Arthroplasty. 2017;32:2717–2724. 28487091 10.1016/j.arth.2017.03.074

[17] Kubo M , Maeda T , Kumagai K , Amano Y , Kawasaki T , Imai S . Good postoperative flexion angle improves knee function and improvement of flexion angle increases patient satisfaction after total knee arthroplasty. J Arthroplasty. 2021;36:3137–3140. 34034923 10.1016/j.arth.2021.04.040

[18] Lewis PL , Graves SE , de Steiger RN , Campbell DG , Peng Y , Hatton A , et al. Does knee prosthesis survivorship improve when implant designs change? Findings from the Australian Orthopaedic Association National Joint Replacement Registry. Clin Orthop Relat Res. 2020;478:1156–1172. 32324669 10.1097/CORR.0000000000001229 PMC7319368

[19] Li G , Most E , Otterberg E , Sabbag K , Zayontz S , Johnson T , et al. Biomechanics of posterior‐substituting total knee arthroplasty: an in vitro study. Clin Orthop Relat Res. 2002;404:214–225. 10.1097/00003086-200211000-00035 12439263

[20] Lustig S , Scholes CJ , Stegeman TJ , Oussedik S , Coolican MRJ , Parker DA . Sagittal placement of the femoral component in total knee arthroplasty predicts knee flexion contracture at one‐year follow‐up. Int Orthop. 2012;36:1835–1839. 22638608 10.1007/s00264-012-1580-z PMC3427457

[21] Lützner C , Beyer F , David L , Lützner J . Fulfilment of patients’ mandatory expectations are crucial for satisfaction: a study amongst 352 patients after total knee arthroplasty (TKA). Knee Surg Sports Traumatol Arthrosc. 2023;31:3755–3764. 36740633 10.1007/s00167-022-07301-y PMC10435619

[22] Matsuda S , Kawahara S , Okazaki K , Tashiro Y , Iwamoto Y . Postoperative alignment and ROM affect patient satisfaction after TKA. Clin Orthop Relat Res. 2013;471:127–133. 22903282 10.1007/s11999-012-2533-y PMC3528933

[23] Nakagawa S , Kadoya Y , Todo S , Kobayashi A , Sakamoto H , Freeman MAR , et al. Tibiofemoral movement 3: full flexion in the living knee studied by MRI. J Bone Joint Surg Br. 2000;82:1199–1200. 11132287 10.1302/0301-620x.82b8.10718

[24] Okamoto Y , Nakajima M , Jotoku T , Otsuki S , Neo M . Capsular release around the intercondylar notch increases the extension gap in posterior‐stabilized rotating‐platform total knee arthroplasty. Knee. 2016;23:730–735. 27174384 10.1016/j.knee.2015.11.022

[25] Quah C , Swamy G , Lewis J , Kendrew J , Badhe N . Fixed flexion deformity following total knee arthroplasty. A prospective study of the natural history. Knee. 2012;19:519–521. 21996572 10.1016/j.knee.2011.09.003

[26] Ritter MA , Lutgring JD , Davis KE , Berend ME , Pierson JL , Meneghini RM . The role of flexion contracture on outcomes in primary total knee arthroplasty. J Arthroplasty. 2007;22:1092–1096. 18078875 10.1016/j.arth.2006.11.009

[27] Rosenberger RE , Hoser C , Quirbach S , Attal R , Hennerbichler A , Fink C . Improved accuracy of component alignment with the implementation of image‐free navigation in total knee arthroplasty. Knee Surg Sports Traumatol Arthrosc. 2008;16:249–257. 18157493 10.1007/s00167-007-0420-y

[28] Scuderi GR , Kochhar T . Management of flexion contracture in total knee arthroplasty. J Arthroplasty. 2007;22:20–24. 10.1016/j.arth.2006.12.110 17570272

[29] Seito N , Onodera T , Kasahara Y , Kondo E , Iwasaki N , Majima T . Preoperative knee deformity and kinematics impact postoperative knee kinematics in total knee arthroplasty. Knee. 2017;24:1462–1468. 28970121 10.1016/j.knee.2017.08.056

[30] Skou ST , Roos EM , Laursen MB , Rathleff MS , Arendt‐Nielsen L , Simonsen O , et al. A randomized, controlled trial of total knee replacement. N Engl J Med. 2015;373:1597–1606. 26488691 10.1056/NEJMoa1505467

[31] Soon EL , Bin Abd Razak HR , Tan CS , Tan HCA . Postoperative range of motion does not correlate with patient reported outcome scores in Asians after total knee arthroplasty. J Arthroplasty. 2014;29:2285–2288. 24656638 10.1016/j.arth.2014.02.018

[32] Sumino T , Tomita T , Sugamoto K , Yamazaki T , Okazaki K . Semi‐constrained posterior stabilized total knee arthroplasty reproduces natural deep knee bending kinematics. BMC Musculoskelet Disord. 2020;21:107. 32066423 10.1186/s12891-020-3059-1 PMC7027226

[33] Tanifuji O , Sato T , Kobayashi K , Mochizuki T , Koga Y , Yamagiwa H , et al. Three‐dimensional in vivo motion analysis of normal knees employing transepicondylar axis as an evaluation parameter. Knee Surg Sports Traumatol Arthrosc. 2013;21:2301–2308. 22543470 10.1007/s00167-012-2010-x

[34] Ueno A , Hashimoto S , Oshima A , Ohsawa T , Takase R , Kaneko S , et al. Postoperative medial tilting of the joint line and preoperative kinematics influence postoperative medial pivot pattern reproduction in total knee arthroplasty. Arthroplast Today. 2023;23:101178. 37712071 10.1016/j.artd.2023.101178 PMC10498393

[35] Yanagisawa S , Sato N , Ohsawa T , Saito K , Shimizu M , Takagishi K . Influence of the anterior‐posterior femoral translation on the range of motion in cruciate‐retaining total knee arthroplasty. Knee Surg Sports Traumatol Arthrosc. 2014;22:2709–2714. 23794004 10.1007/s00167-013-2579-8

[36] Zhou Z , Yew KSA , Arul E , Chin PL , Tay KJD , Lo NN , et al. Recovery in knee range of motion reaches a plateau by 12 months after total knee arthroplasty. Knee Surg Sports Traumatol Arthrosc. 2015;23:1729–1733. 25178534 10.1007/s00167-014-3212-1

